# Limited Diagnostic and Therapeutic Value of Chest X-Rays in Hematological Patients With Febrile Neutropenia

**DOI:** 10.1093/ofid/ofaf419

**Published:** 2025-07-18

**Authors:** Dorine Dijkshoorn-Fokker, Madalina Marina, Ada van Bruchem-van de Scheur, Wendy Oldenmenger, Bart Rijnders, Jurjen Versluis, Nick Wlazlo

**Affiliations:** Department of Hematology, Erasmus University Medical Center Cancer Institute, Rotterdam, The Netherlands; Department of Hematology, Erasmus University Medical Center Cancer Institute, Rotterdam, The Netherlands; Master Advanced Nursing Practice, Rotterdam University of Applied Sciences, Rotterdam, The Netherlands; Department of Medical Oncology, Erasmus University Medical Center Cancer Institute, Rotterdam, The Netherlands; Department of Medical Microbiology and Infectious Disease, Erasmus University Medical Center, Rotterdam, The Netherlands; Department of Hematology, Erasmus University Medical Center Cancer Institute, Rotterdam, The Netherlands; Department of Hematology, Erasmus University Medical Center Cancer Institute, Rotterdam, The Netherlands

**Keywords:** chest X-ray, CT (Computed tomography) scan, febrile neutropenia, hematologic malignancies

## Abstract

**Background:**

In hematological patients with febrile neutropenia, chest X-rays are frequently performed to exclude possible pulmonary infections. However, the diagnostic and therapeutic value of this imaging remains unclear.

**Methods:**

We conducted a retrospective observational cohort study over a 2-year period, examining episodes of febrile neutropenia in adult patients treated with myelosuppressive chemotherapy. Febrile episodes were categorized based on the presence (group A) or absence (group B) of respiratory symptoms. We assessed the frequency of abnormal chest X-rays and chest computed tomography (CT) scans and their impact on antimicrobial treatment decisions.

**Results:**

Of the 412 febrile episodes in 259 patients, 41.4% in group A and 16.1% in group B had an abnormal chest X-ray (*P* < .001). X-rays showing infiltrates were followed by chest CT in 51.5%. Antimicrobial treatment decisions were rarely based on results of X-rays: 6.9% (95% confidence interval [CI], 2.4%–15.6%) in group A and 3.4% (95% CI, 1.9%–5.7%) in group B (*P* = .200). In group A, however, antimicrobial treatment was more often adjusted based on an ensuing abnormal CT: 17.2% (95% CI, 9.2%–28.4%) versus 6.2% (95% CI, 4.0%–9.1%) (*P* = .004).

**Conclusions:**

Chest X-rays rarely influence antibiotic treatment decisions in febrile neutropenia and can probably be safely omitted, especially in patients without respiratory symptoms.

Febrile neutropenia is a common complication in patients receiving myelosuppressive chemotherapy for hematologic malignancies [1]. This is particularly prevalent in patients with acute myeloid leukemia (AML), with incidence rates exceeding 80%. Fever in these patients might be the first and only sign of a severe, life-threatening infection. A source of infection is identified in approximately 20%–30% of fever episodes, and 4%–6% of patients die from bacterial infections [2]. Additionally, fungal infections are a significant cause of neutropenic fever, with about 10% of patients experiencing invasive fungal infections [3]. As part of the diagnostic workup, including a comprehensive history and physical examination, blood cultures (peripheral and central line) are obtained, and antibiotic treatment should be started immediately. To investigate a pulmonary cause of the fever, chest X-rays are frequently performed in the workup of febrile neutropenia. The European Society for Medical Oncology recommends chest X-rays for all adult patients with neutropenic fever [4], while the Infectious Diseases Society of America recommends a chest X-ray only in patients with respiratory symptoms [5].

Retrospective studies have shown that the likelihood of abnormal findings on a chest X-ray in hematological patients without respiratory symptoms is very low, ranging from 0 to 5%. In contrast, hematological patients with clinical respiratory symptoms have a higher incidence of abnormal chest X-rays, ranging from 16% to 32% [6–8]. On the other hand, there is abundant literature that chest computed tomography (CT) scans are superior to chest X-rays in detecting both bacterial pneumonia and fungal infections. In this regard, chest X-rays have a lower sensitivity, ranging from 18% to 50%, compared to CT scans, ranging from 63% to 91% [3, 6, 9–12].

There has been limited research on the impact of abnormal chest radiograph findings on further diagnostic investigations and the management of febrile neutropenia, particularly in patients with prolonged neutropenia. This study aims to investigate the diagnostic yield of routine chest X-rays and follow-up diagnostics, including chest CT, in clinical practice. Additionally we aim to determine how frequently chest X-ray findings have influenced the management and treatment of febrile neutropenia in patients with AML and recipients of allogeneic stem cell transplantation (SCT). We also investigated whether a significant difference can be demonstrated between febrile episodes with and without respiratory symptoms.

## METHODS

### Patients

We performed a retrospective cohort study including all patients aged 18 years and older admitted to the Hematology Department of the Erasmus Medical Center, Rotterdam, the Netherlands, between 1 September 2020 and 31 August 2022, who were treated with intensive myelosuppressive chemotherapy for AML or received conditioning for allogeneic SCT. All patients received antimicrobial prophylaxis with ciprofloxacin and fluconazole during neutropenia. We collected data on all episodes of febrile neutropenia in which a chest X-ray was performed within 48 hours of onset. As such, data from multiple fever episodes could be collected for a single patient. A new fever episode was defined when it occurred at least 5 days after the start of the previous episode and there was an interval of at least 24 hours with a normal temperature between episodes. Febrile neutropenia was protocol-defined as a single temperature of >38.7°C or a temperature measured twice at 1-hour intervals of >38.2°C, both in the presence of absolute neutrophil count <0.5 × 10^9^ cells/L. Patients with febrile neutropenia were typically treated with broad-spectrum antibiotics such as piperacillin/tazobactam or meropenem during the relevant period. Antibiotics were always discontinued after 72 hours if no bacterial infection was diagnosed after taking blood cultures, urine cultures, and routine chest X-ray [13]. Patients diagnosed with (hospital-) acquired bacterial pneumonia were treated for 5–7 days in our protocol. Patients with other infectious diseases (eg, catheter-related central bloodstream infections) were treated according to local guidelines. In patients with persistent neutropenic fever after 5 days, local policy was to perform a chest CT. Other additional diagnostics were performed at the discretion of the medical team. The study was approved by the Medical Ethical Committee of our hospital.

### Data Collection

For each episode of febrile neutropenia, baseline data were collected regarding age, sex, hematological disease and current treatment, history of chronic obstructive pulmonary disease and asthma, leukocyte count, and in particular the presence of respiratory symptoms including dyspnea, cough, and/or sputum on day 1 and at the time of the chest CT imaging. Both reports and images of chest X-rays and subsequent chest CT scans were reviewed by an independent radiologist and 2 independent researchers (D. D. and N. W.) for abnormalities such as pulmonary infiltrates, nodules, pleural effusions, ground glass opacities, cardiomegaly, and other findings (atelectasis, lymphadenopathy, emphysema, aspecific micronoduli, thrombus). Abnormalities were classified as such if they were new compared to previous imaging, or if no previous imaging was available. Multiple abnormalities could be found per chest X-ray or CT scan. In addition, the results of subsequently performed bronchoalveolar lavage (BAL) were collected. Finally, data were collected on treatment, considering type and duration of initial broad-spectrum antibiotics, antifungals, and changes thereafter, as well as 30-day mortality.

A change in antimicrobial policy was defined as dosing broad-spectrum antibiotics for longer than the above-mentioned 72 hours, starting or changing treatment to other antibiotics, or starting antifungal drugs. Two researchers (D. D. and N. W.) determined whether antimicrobial policy had changed within the first 7 days of the febrile episode as a result of findings on chest X-ray, chest CT, other imaging (eg, abdominal CT, magnetic resonance imaging), and/or as a result of other microbiological testing (ie, cultures, *Aspergillus* antigen testing, *Aspergillus*/mucormycosis polymerase chain reaction), using a predefined protocol. Multiple factors (imaging, cultures) could therefore determine the treatment change. When chest CT scans were already performed during the first 72 hours of antibiotic treatment and treatment was prolonged to treat bacterial pneumonia, episodes were classified as “changed treatment policy based on CT,” as CT scan findings override chest X-ray findings in practice. To investigate the combined benefits of a chest X-ray and CT scan in the first 72 hours, we also constructed a variable of antimicrobial treatment adjustment “based on combined X-ray and CT scan findings within the first 72 hours.” In case of disagreement between the 2 reviewers, cases and images were discussed until agreement was reached.

### Data Analysis

The quantitative data were collected and analyzed using the statistical software program SPSS, version 28. Descriptive statistics were used to provide insight into demographic and medical variables. Categorical variables were represented as frequencies and percentages. Continuous variables were presented as means and standard deviations or as median with interquartile range when not normally distributed. The febrile neutropenia episodes were divided into fever episodes with respiratory symptoms (group A) and fever episodes without respiratory symptoms (group B). Comparisons between these groups were made using the χ^2^ test when comparing categorical variables between group A and B, and the *t* test and the Mann-Whitney *U* test for continuous variables. Finally, a sensitivity analysis was performed using a narrower definition of group A as episodes of “clinically suspected bacterial pneumonia” with dyspnea or sputum in addition to cough symptoms, and a group B including episodes of dry cough only. The results were considered statistically significant if *P* < .05.

## RESULTS

### Baseline Characteristics

A total of 259 patients were identified who received intensive myelosuppressive chemotherapy for AML or conditioning for allogeneic SCT. Febrile neutropenia followed by a chest X-ray was observed in 198 (77.4%) patients, where 412 episodes were found corresponding with 1.6 episodes per patient. Fifty-eight episodes (14.1%) were accompanied by 1 or more respiratory symptoms and were categorized into group A. Sixteen episodes (3.9%) had a combination of symptoms that was clinically suspicious for bacterial pneumonia. Patient characteristics and details of the febrile episodes are described in Table 1.

**Table 1.** Background Characteristics of the Study Population and Details of Fever Episodes

Characteristic	Outcome
No. of patients with neutropenic fever	n = 198
Age, y, mean (SD)	54.9 (14.4)
Sex	
Male	116 (58.6)
Female	82 (41.4)
Hematological disease	
Acute myeloid leukemia	110 (55.6)
Acute lymphatic leukemia	21 (10.6)
Myelodysplastic syndrome	22 (11.1)
Non-Hodgkin lymphoma	15 (7.6)
Myeloproliferative neoplasms	7 (3.5)
Other	23 (11.6)
History of COPD and/or asthma	10 (5.1)
Febrile neutropenia episodes	n = 412
Hematological disease	
Acute myeloid leukemia	271 (65.8)
Acute lymphatic leukemia	32 (7.8)
Myelodysplastic syndrome	48 (11.6)
Non-Hodgkin lymphoma	19 (4.6)
Myeloproliferative neoplasms	7 (1.7)
Other	35 (8.5)
Hematological treatment	
Myelosuppressive chemotherapy	251 (60.9)
Allogeneic stem cell transplantation	161 (39.1)
Leukocyte count at start of fever, ×10^9^/L, median (IQR)	0.22 (0.08–0.48)
Respiratory symptoms on day 1	58 (14.1)
Dyspnea	9 (2.2)
Coughing	48 (11.7)
Sputum	9 (2.2)
Clinical pneumonia (dyspnea, coughing with sputum)	16 (3.9)

Data are presented as No. (%) unless otherwise indicated.

Abbreviations: COPD, chronic obstructive pulmonary disease; IQR, interquartile range; SD, standard deviation.

### Chest X-Ray and CT Findings

**Figure 1.**
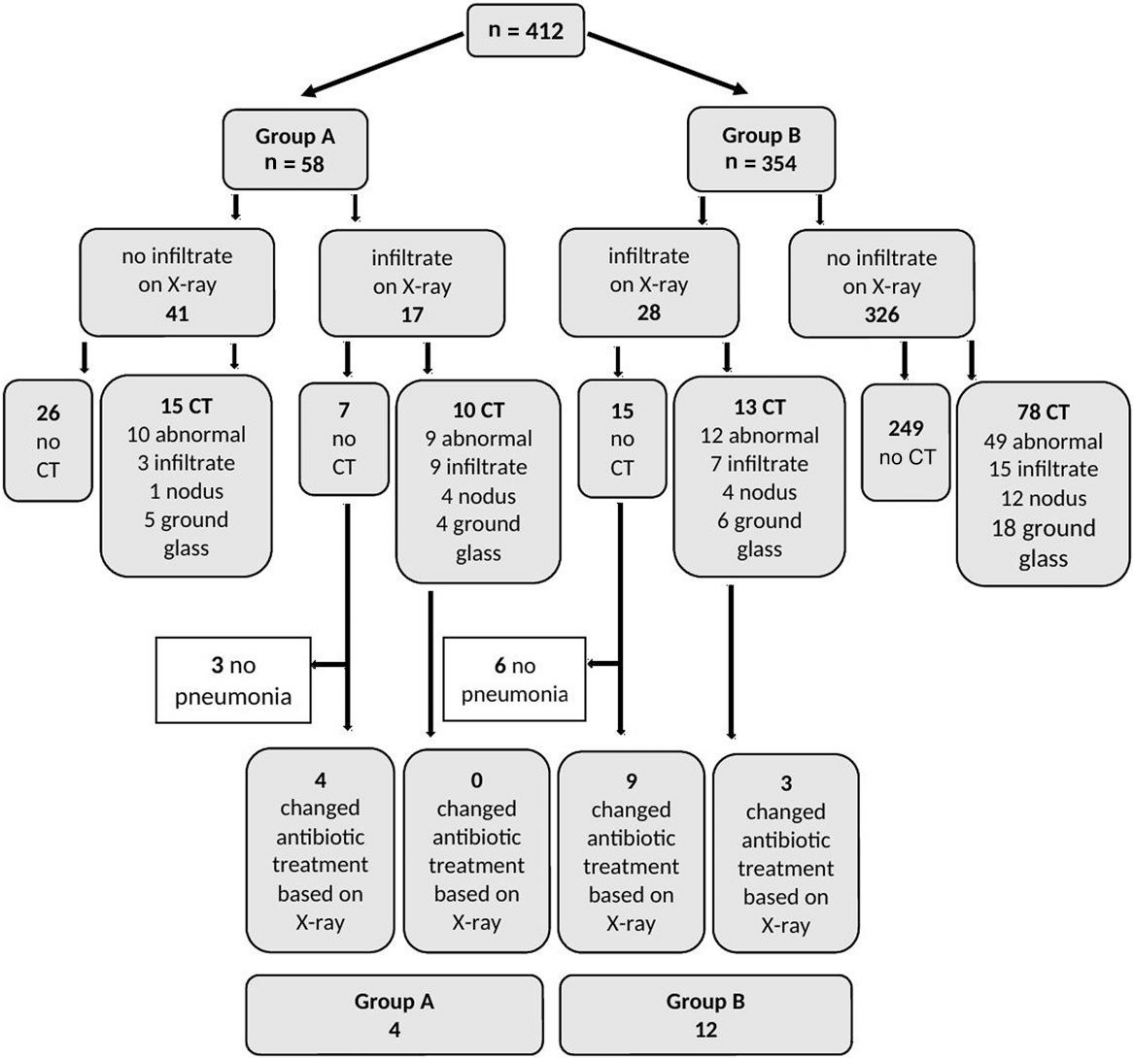
Flowchart of impact of chest X-ray on treatment policy in group A (respiratory symptoms) and group B (no respiratory symptoms). Abbreviation: CT, computed tomography.

Figure 1 shows a flowchart of the diagnostic investigations performed in group A and group B. Overall, 81 (19.7%) chest X-rays showed new abnormalities, with pulmonary infiltrates detected on 45 (10.9%) chest X-rays (Table 2). Abnormal chest X-rays were more frequent in patients presenting with respiratory symptoms (group A) compared with those without symptoms (group B): 41.4% (95% confidence interval [CI], 29.4%–54.2%) versus 16.1% (95% CI, 12.6%–20.2%) (*P* < .001). The majority of these findings were pulmonary infiltrates, seen more frequently in patients with respiratory symptoms: 29.3% (95% CI, 18.8%–41.8%) in group A versus 7.9% (95% CI, 5.4%–11.1%) in group B (*P* < .001).

**Table 2.** Abnormal Chest X-Ray, Chest Computed Tomography, and Bronchoalveolar Lavage in Group A (With Respiratory Symptoms) and Group B (Without Respiratory Symptoms)

Findings on Chest X-Ray, CT Scan, and BAL	Total (N = 412)	Group A (n = 58)	Group B (n = 354)	*P* Value
Chest X-ray unremarkable	331 (80.3)	34 (58.6)	297 (83.9)	<.001
Chest X-ray abnormal	81 (19.7)	24 (41.4)	57 (16.1)	<.001
Infiltrate	45 (10.9)	17 (29.3)	28 (7.9)	<.001
Nodus	0 (0.0)	0 (0.0)	0 (0.0)	
Pleural fluid	24 (5.8)	5 (8.6)	19 (5.4)	.327
Cardiomegaly	7 (1.7)	1 (1.7)	6 (1.7)	.987
Other	25 (6.1)	8 (13.8)	17 (4.8)	.008
CT scan performed	116 (28.2)	25 (43.1)	91 (25.7)	.006
CT scan abnormal	80 (19.4)	19 (32.8)	61 (17.2)	.006
Infiltrate	34 (8.3)	12 (20.7)	22 (6.2)	<.001
Noduli	21 (5.1)	5 (8.6)	16 (4.5)	.188
Ground glass	33 (8.0)	9 (15.5)	24 (6.8)	.023
Pleural fluid	29 (7.0)	9 (15.5)	20 (5.6)	.006
Cardiomegaly	1 (0.2)	1 (1.7)	0 (0.0)	.013
Other	34 (8.3)	7 (12.1)	27 (7.6)	.254
BAL performed	22 (5.3)	7 (12.1)	15 (4.2)	.014
Microbiological evidence of fungal infection (IPA/mucormycosis)	8 (1.9)	1 (1.7)	7 (2.0)	.897

Data are presented as No. (%) unless otherwise indicated.

Abbreviations: BAL, bronchoalveolar lavage; CT, computed tomography; IPA, invasive pulmonary aspergillosis.

Chest CT scans were performed in 116 (28.2%) fever episodes. The majority of these episodes were characterized by persistent fever or respiratory symptoms at the time of CT (n = 103 [88.8%]). More chest CT scans were performed in group A than in group B: 43.1% (95% CI, 31.0%–55.9%) versus 25.7% (95% CI, 21.4%–30.4%) (*P* = .006). We observed that febrile episodes with a pulmonary infiltrate detected on chest X-rays were followed by subsequent chest CT in 51.1% (23 of 45; Figure 1). In addition, chest CT scans were performed in 25.3% (93 of 367; Figure 1) of febrile episodes without infiltrates on chest X-rays. The majority of chest CT scans detected abnormalities (69.0%; Table 2). Pulmonary infiltrates were significantly more common in group A than in group B: 20.7% (95% CI, 11.8%–32.4%) versus 6.2% (95% CI, 4.0%–9.1%) (*P* < .001), and also ground glass opacities: 15.5% (95% CI, 8.0%–26.4%) versus 6.8% (95% CI, 4.5%–9.8%) (*P* = .023). However, the presence of nodular infiltrates suggestive of an invasive fungal infection was not significantly higher in groups A and B: 8.6% (95% CI, 3.4%–17.9%) versus 4.5% (95% CI, 2.7%–7.1%) (*P* = .188). BAL was performed in 5.3% of febrile episodes, and an invasive fungal infection (invasive pulmonary aspergillosis/mucormycosis) was detected in 1.9%. BAL was performed more frequently in group A than in group B (12.1% vs 4.2%; *P* = .014), but the microbiological evidence for a pulmonary fungal infection was equally frequent in both groups (1.7% vs 2.0%; *P* = .897).

### Impact of Chest X-Ray on Antimicrobial Treatment

In 98.5% of febrile episodes, a broad-spectrum antibiotic was started at the onset of fever: piperacillin/tazobactam (52.2%), meropenem (45.4%), or meropenem with vancomycin (1%). During 16 episodes (3.8%), antifungal agents for invasive aspergillosis or mucormycosis were started, of which 8 were substantiated by microbiological evidence from a BAL (Table 3). Even though more episodes in group A (8.6%) were treated with antifungal agents than in group B (3.1%), the incidence of a probable invasive fungal infection was similar (Table 2).

**Table 3.** Antibiotic/Antifungal Treatments and Adjustments in Group A (With Respiratory Symptoms) and Group B (Without Respiratory Symptoms) and Mortality Rate Within 30 Days After a Fever Episode

Treatment	Total (N = 412)	Group A (n = 58)	Group B (n = 354)	*P* Value
Broad-spectrum antibiotic started	406 (98.5)	56 (96.6)	350 (98.9)	.088
Length of antibiotic therapy, d, median (IQR)	3.0 (3.0–5.0)	3.0 (3.0–7.0)	3.0 (3.0–3.0)	<.001
Antifungal treatment started	16 (3.8)	5 (8.6)	11 (3.1)	.044
Antibiotic treatment changed	205 (49.8)	33 (56.9)	172 (48.6)	.241
Based on X-ray findings alone	16 (3.9)	4 (6.9)	12 (3.4)	.200
Based on combined X-ray and CT scan findings <72 h	28 (6.8)	10 (17.2)	18 (5.1)	<.001
Based on CT scan findings	32 (7.8)	10 (17.2)	22 (6.2)	.004
Based on other imaging findings	26 (6.3)	6 (10.3)	20 (5.6)	.173
Based on results of microbiological examinations	147 (35.7)	16 (27.6)	131 (37.0)	.165
Mortality <30 d after fever onset	23 (11.6)	6 (10.3)	17 (4.8)	.088

Data are presented as No. (%) unless otherwise indicated.

Abbreviations: CT, computed tomography; IQR, interquartile range; IPA, invasive pulmonary aspergillosis.

Changes in antimicrobial treatment during the febrile episodes occurred in 49.8% of all fever episodes and were similar between both groups: 56.9% (95% CI, 44.1%–69.0%) for group A and 48.6% (95% CI, 43.4%–53.8%) for group B (*P* = .241). Overall, antimicrobial treatment was mostly adjusted based on results of microbiological tests (35.7%), such as blood cultures, followed by adjustments based on results of chest CT (7.8%) and other imaging modalities (6.3%). Antimicrobial treatment was changed because of findings on chest X-rays in 3.9% (95% CI, 2.3%–6.1%) only. Although in group A antibiotic treatment was changed slightly more often based on chest X-ray results, this was not significantly different from group B: 6.9% (95% CI, 2.4%–15.6%) versus 3.4% (95% CI, 1.9%–5.7%) (*P* = .200). On the other hand, antimicrobial treatment was changed significantly more often based on CT findings in patients with respiratory symptoms compared with those without respiratory symptoms: 17.2% (95% CI, 9.2%–28.4%) versus 6.2% (95% CI, 4.0%–9.1%) (*P* = .004).

This low number of antimicrobial treatment changes based on chest X-ray was related to the fact that 20 of 45 episodes with pulmonary infiltrates on X-ray were followed by chest CT within 72 hours of the febrile episode, and therefore results of the CT scan determined treatment decisions during the first 72 hours of empirical antibiotic therapy (Figure 1). When combining findings of chest X-ray and CT scans within the first 72 hours, the yield in group A increased to 17.2% (95% CI, 9.2%–28.4%), although the absolute gain was from 4 to 10 episodes. In group B, the combined yield remained low: 5.1% (95% CI, 3.2%–7.7%). Another explanation was that 9 of 45 pulmonary infiltrates that were reported by the radiologist on the chest X-ray, were not considered clinically relevant and were therefore not treated as bacterial pneumonia by the medical team.

Finally, the mortality rate within 30 days of initiation of a febrile neutropenia episode was 10.3% (95% CI, 3.9%–21.2%) in group A and 4.8% (95% CI, 2.8%–7.6%) in group B (*P* = .088).

### Sensitivity Analysis

**Figure 2.**
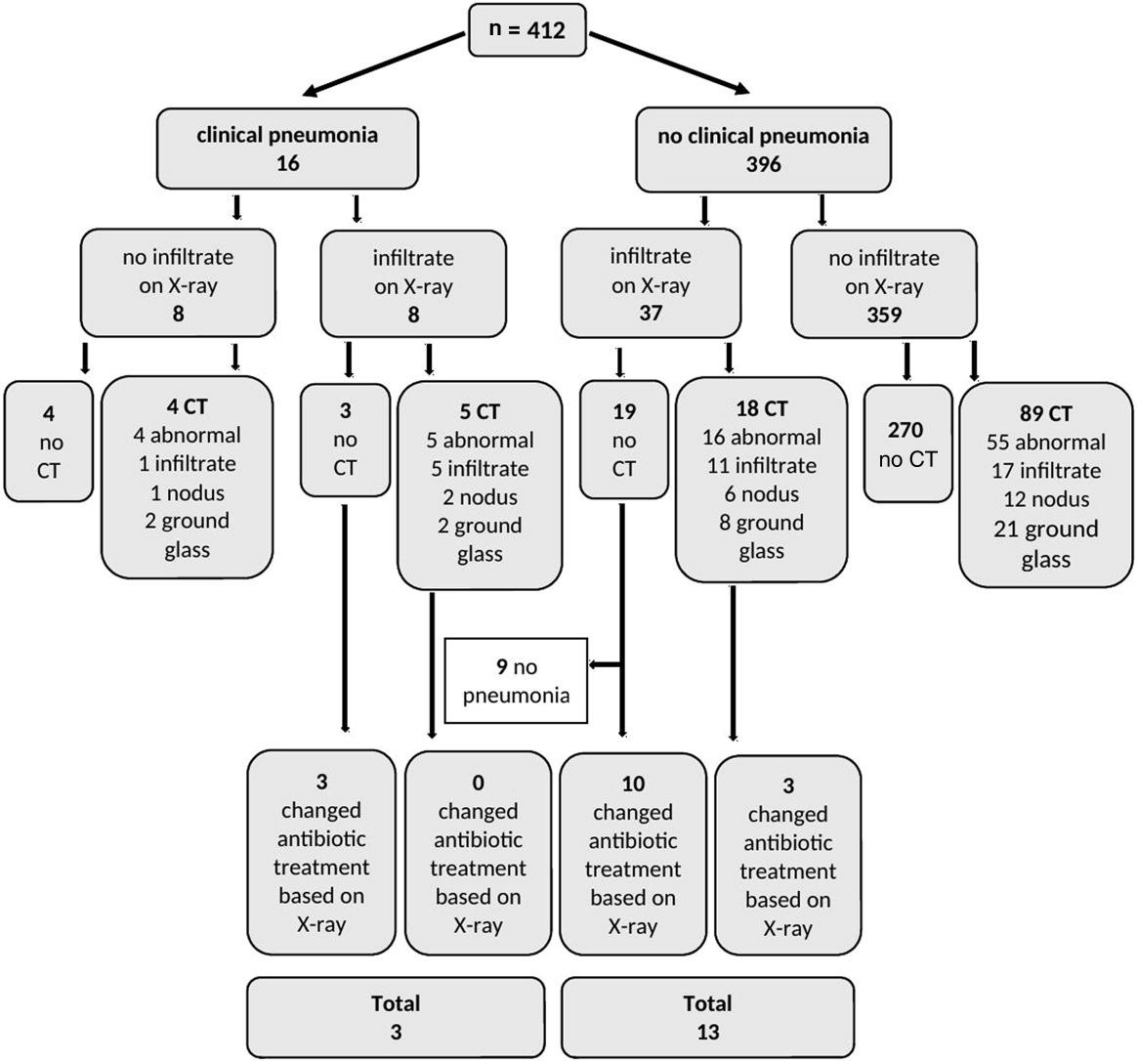
Flowchart of impact of chest X-ray on treatment policy in clinical pneumonia (sensitivity analysis). Abbreviation: CT, computed tomography.

Finally, we performed a sensitivity analysis in which group A (n = 16) was more strictly defined as “clinically suspected bacterial pneumonia” based on the presence of febrile neutropenia together with either dyspnea or the presence of productive cough/sputum (Figure 2). As such, group B also included febrile episodes with nonproductive cough symptoms (n = 396). This analysis showed that in group B, only 3.3% (95% CI, 1.9%–5.4%) of febrile episodes had a change in antimicrobial treatment based on chest X-ray findings, similar to the primary analysis: 3.4% (95% CI, 1.9%–5.7%). The impact of the chest CT scans remained unchanged in group B in the sensitivity analysis: 6.8% (95% CI, 4.6%–9.6%) versus 6.2% (95% CI, 4.0%–9.1%), as did the impact of combined chest X-ray and CT within 72 hours (data not shown).

In the small group of febrile episodes with clinically suspected bacterial pneumonia (group A, n = 16), treatment was adjusted in 18.8% (95% CI, 5.6%–42.1%) because of findings on chest X-rays versus 6.9% (95% CI, 2.4%–15.6%) in primary analysis. However, 56.2% of these episodes had a consecutive chest CT, and treatment was more often adjusted based on this CT: 31.3% (95% CI, 13.1%–55.6%) versus 17.2% (95% CI, 9.2%–28.4%) in primary analysis.

## DISCUSSION

This retrospective observational cohort study in patients with a hematological malignancy with febrile neutropenia suggests that a routinely performed chest X-ray had limited diagnostic and therapeutic value. The detection rate was low, and (abnormal) chest X-rays were often followed by chest CT scans in our practice. Although febrile episodes with respiratory symptoms were more often accompanied by pulmonary infiltrates, we observed that therapeutic changes in antimicrobial management were rarely influenced by findings on routine chest X-rays at the onset of febrile neutropenia.

Although routine chest X-rays detected relatively few abnormalities overall, they revealed a pulmonary infiltrate in 29.3% of patients with respiratory symptoms and 7.9% in patients without respiratory symptoms. These frequencies are in line with what has been previously observed in retrospective studies: namely, 0–5% in patients without respiratory symptoms and 16%–32% in patients with respiratory symptoms [6–8]. However, the proportion of patients *with* respiratory symptoms is small in most of the studies, and so the majority of chest X-rays are of little use.

It is well known that chest CT scans are superior to chest X-rays in detecting both pneumonia and fungal infection [3, 6, 9–12]. Abnormalities on chest CT scans were found twice as often in group A than in group B, especially pulmonary infiltrates, ground glass opacities, and pleural effusions. However, the incidence of a possible or probable invasive fungal infection as suggested by pulmonary nodules, halo signs, or cavitating lesions on CT was similar in both groups. Therefore, the presence of respiratory symptoms does not increase the likelihood of an invasive fungal infection. Furthermore, we observed that an abnormal chest X-ray was often followed by chest CT scan within days (51.1%) to further characterize the abnormalities. On the other hand, most CT scans were performed because of persistent fever and/or respiratory symptoms (88.8%). When chest CT scans are used for these indications, the usefulness of the routine chest X-ray becomes questionable.

There is scarce literature regarding the therapeutic implications of chest X-rays. Two retrospective studies suggested a very limited influence, but these studies have not reported on antimicrobial treatment changes in such detail [7, 8]. This study is the first to show that only rarely (3.9% of febrile episodes) was antimicrobial treatment adjusted because of abnormal findings on a chest X-ray, and that this finding was independent of the presence of respiratory symptoms. The main reasons for this low value were that many chest X-rays that showed pulmonary infiltrates were quickly followed by chest CT or were not interpreted as bacterial pneumonia by the medical team. Our data therefore suggest that a routine chest X-ray will have limited impact on the antimicrobial therapy. We confirmed the important role of microbiological examinations in both groups, leading to changes in antimicrobial treatment in one-third of fever episodes. Moreover, we showed that in febrile episodes with respiratory symptoms or with clinically suspected pneumonia (sensitivity analysis), findings from chest CT scans (whether or not preceded by chest X-ray) led to changes in antimicrobial treatment in 17% and 31%, respectively. It may therefore be a more efficient use of resources to leave the chest X-ray out of the diagnostic approach and to perform a chest CT in those cases with clinically suspected pneumonia or when unexplained fever persists 72 hours after the start of empirical antibiotic therapy. Ideally, both such strategies would be compared in a randomized trial to confirm this conclusion, as our study measured clinician behavior rather than whether antimicrobial prescribing is appropriate in all cases. Since respiratory infiltrates can represent infection requiring antibiotics, infection not requiring antibiotics (eg, viral infection), or noninfectious causes (eg, sterile aspiration, drug-induced pneumonitis), we do not always know that identification of more abnormalities on CT is superior in all cases.

A strong point of this study is that it showed that chest X-rays have limited value in febrile episodes during prolonged neutropenia on myelosuppressive chemotherapy and allogeneic SCT. As such, the results are in line with previous studies in patients undergoing less intensive treatments or autologous SCT. In the very small group of patients with a strong clinical suspicion of pneumonia or pulmonary infection, the yield of chest X-rays is a little higher, but it seems more logical to perform chest CT scans in this group.

One of the limitations of this retrospective study is the potential for confounding and measurement error, as data that may have been related to the research question were not systematically recorded. This was the case for reporting the presence or absence of respiratory symptoms during the onset of neutropenic fever and the reasons for performing a CT scan or not. In the absence of blinding, the retrospective nature may introduce bias in the interpretation of whether a chest X-ray led to an adjustment in antimicrobial treatment. We tried to minimize this by independently assessing all abnormal chest X-rays, CT scans, and the variables related to treatment changes by 2 researchers according to uniform criteria in the research protocol. An inherent limitation is that we could not prove *whether* antibiotic adjustments were justified in patients with infiltrates but without positive microbiological studies. With a low incidence of viral pneumonia in hospitalized patients with AML and/or SCT, it is, however, common practice to treat such patients with prolonged antibiotic therapy for bacterial pneumonia. Moreover, if some of the changes in antimicrobial policy would have been unnecessary, the yield of changes in antimicrobial policy prompted by chest X-ray findings would have been even lower than the 3.9% observed. Finally, we did not adjust statistically for the inclusion of multiple febrile episodes per patient. Since we defined new neutropenic fever episodes widely (ie, 7 days apart), we believe that these could be considered independent fever episodes.

## CONCLUSIONS

In hematological patients with febrile neutropenia during intensive myelosuppressive chemotherapy or allogeneic SCT, changes in antimicrobial treatment were rarely based on findings on routine chest X-rays. We therefore suggest that, in the absence of convincing data proving the opposite, a chest X-ray can be safely omitted from the routine workup, at least in patients without clinical suspicion of pneumonia. Omitting a chest X-ray leads to more efficient care, with less (radiation) burden and infection risk for the patient and a cost and capacity savings for the organization.

## Notes

***Author contributions.*** D. D. and N. W. designed the research. D. D. performed the research and collected data. D. D. and N. W. evaluated all radiological imaging, allocated treatment changes, and performed statistical analyses. All authors interpreted the data and contributed to writing of the manuscript.

## References

[1] Stichting Werkgroep Antibioticabeleid . Guideline: recommendations for the diagnosis and management of neutropenic fever in patients with cancer. **2022**. Available at: https://swab.nl/nl/exec/file/download/186. Accessed September 5, 2023.

[2] Rivas-Ruiz R, Villasis-Keever M, Miranda-Novales G, Castelán-Martínez OD, Rivas-Contreras S. Outpatient treatment for people with cancer who develop a low-risk febrile neutropaenic event. Cochrane Database Syst Rev 2019; 3:CD009031. 30887505 10.1002/14651858.CD009031.pub2 PMC6423292

[3] Gerritsen MG, Willemink MJ, Pompe E, et al Improving early diagnosis of pulmonary infections in patients with neutropenic fever using low-dose chest computed tomography. PLoS One 2017; 12:e0172256. 28235014 10.1371/journal.pone.0172256 PMC5325310

[4] Klastersky J, de Naurois J, Rolston K, et al Management of febrile neutropaenia: ESMO clinical practice guidelines. Ann Oncol 2016; 27(Suppl 5):v111–8. 27664247 10.1093/annonc/mdw325

[5] Freifeld AG, Bow EJ, Sepkowitz KA, et al Clinical practice guideline for the use of antimicrobial agents in neutropenic patients with cancer: 2010 update by the Infectious Diseases Society of America. Clin Infect Dis 2011; 52:e56–93. 21258094 10.1093/cid/cir073

[6] El Majzoub I, El Zakhem A, Cheaito R, et al The utility of chest X-ray vs. computed tomography in neutropenic fever patients presenting to the emergency department. J Infect Dev Ctries 2020; 14:1178–84. 33175714 10.3855/jidc.12577

[7] Estacio O, Loh Z, Baker A, et al Limited utility of routine chest X-ray in initial evaluation of neutropenic fever in patients with haematological diseases undergoing chemotherapy. Intern Med J 2018; 48:556–60. 29227565 10.1111/imj.13712

[8] Yolin-Raley DS, Dagogo-Jack I, Niell HB, et al The utility of routine chest radiography in the initial evaluation of adult patients with neutropenic fever patients undergoing HSCT. J Natl Compr Canc Netw 2015; 13:184–9. 25691611 10.6004/jnccn.2015.0027

[9] Kim HJ, Park SY, Lee HY, Lee KS, Shin KE, Moon JW. Ultra-low-dose chest CT in patients with neutropenic fever and hematologic malignancy: image quality and its diagnostic performance. Cancer Res Treat 2012; 46:393–402. 10.4143/crt.2013.132 PMC4206072 25308150

[10] Stanzani M, Sassi C, Lewis R, et al Early low-dose computed tomography with pulmonary angiography to improve the early diagnosis of invasive mould disease in patients with haematological malignancies: a pilot study. J Infect 2021; 83:371–80. 34171366 10.1016/j.jinf.2021.06.019

[11] Burivong W, Sricharoen T, Thachang A, Soodchuen S, Maroongroge P, Leelasithorn V. Early radiologic diagnosis of pulmonary infection in febrile neutropenic patients: a comparison of serial chest radiography and single CT chest. Radiol Res Pract 2021; 2021:8691363. 33680511 10.1155/2021/8691363 PMC7906812

[12] Schalekamp S, Van Ginneken B, Van den Berk IA, et al Bone suppression increases the visibility of invasive pulmonary aspergillosis in chest radiographs. PLoS One 2014; 9:e108551. 25279774 10.1371/journal.pone.0108551 PMC4184785

[13] Schauwvlieghe A, Dunbar A, Storme E, et al Stopping antibiotic therapy after 72 h in patients with febrile neutropenia following intensive chemotherapy for AML/MDS (safe study): a retrospective comparative cohort study. EClinicalMedicine 2021; 35:100855. 33997746 10.1016/j.eclinm.2021.100855 PMC8099620

